# Managing two cases of twin anemia–polycythemia sequence in monochorionic twin pregnancies

**DOI:** 10.3389/fmed.2024.1504772

**Published:** 2025-01-28

**Authors:** Yongke Zhang, Yiheng Liang, Ran Chen, Dirong Zhang, Jing Wu

**Affiliations:** ^1^Department of Obstetrics and Gynecology, Peking University Shenzhen Hospital, Shenzhen, China; ^2^Department of Ultrasonography, Peking University Shenzhen Hospital, Shenzhen, China; ^3^Medical Genetic Center, Guangdong Women and Children Hospital, Guangzhou, China

**Keywords:** monochorionic twin pregnancy, twin anemia-polycythemia sequence, middle cerebral artery peak systolic velocity, fetoscopic laser therapy, ultrasound monitoring

## Abstract

**Background:**

Twin anemia–polycythemia sequence (TAPS) is a rare monochorionic complication. In this study, we discuss the management of two cases of TAPS with different conditions.

**Case presentation:**

Patient 1 was a 34-year-old multigravida in whom the fetal middle cerebral artery peak systolic velocities (MCA-PSVs) were 0.86 multiples of the median (MoM) and 2.0 MoM at 33 weeks of gestation. After cesarean section, stage 3 TAPS was confirmed according to the ultrasound findings and hemoglobin results of the newborns and placenta examination after birth. Patient 2 was a nulligravida who was diagnosed with stage 2 TAPS at 18 weeks of gestation. The patient underwent fetoscopic laser surgery. Ultrasonography monitoring of the MCA-PSVs was performed on a schedule after surgery, with a good status but selective intrauterine growth restriction of one cotwin. The newborns reached their normal development milestones after spontaneous preterm birth.

**Conclusion:**

Optimal management should be carefully selected for patients with different TAPS conditions.

## Introduction

Twin anemia–polycythemia sequence (TAPS) is a rare monochorionic complication that can be divided into two categories: postlaser TAPS, which is a complication of laser surgery for twin-twin transfusion syndrome (TTTS), and spontaneous TAPS. Postlaser TAPS occurs in 2–13% of TTTS pregnancies treated with laser ablation, up to 6 weeks after the procedure (1). Most cases of spontaneous TAPS are discovered in the third trimester in monochorionic twins, with an incidence of 3–6% (2). The pathogenesis of TAPS remains unknown (3) but is typically attributed to unidirectional arteriovenous (A-V) anastomoses, which are usually very small (<1 mm), without accompanying arterioarterial (A-A) anastomoses. The small residual anastomoses allow slow passage of red cells from the donor twin to the recipient twin, gradually leading to highly discordant hemoglobin levels (1). The recipient twin becomes polycythemic, whereas the donor twin becomes anemic (4). Hemodynamic compensation protects the amniotic fluid volume discordancy, thus distinguishing it from TTTS (5). Severe polycythemia can lead to fetal and placental thrombosis, whereas severe anemia can lead to hydrops fetalis or the death of one or both fetuses (6).

## Case presentation

### Patient 1

**Figure 1:**
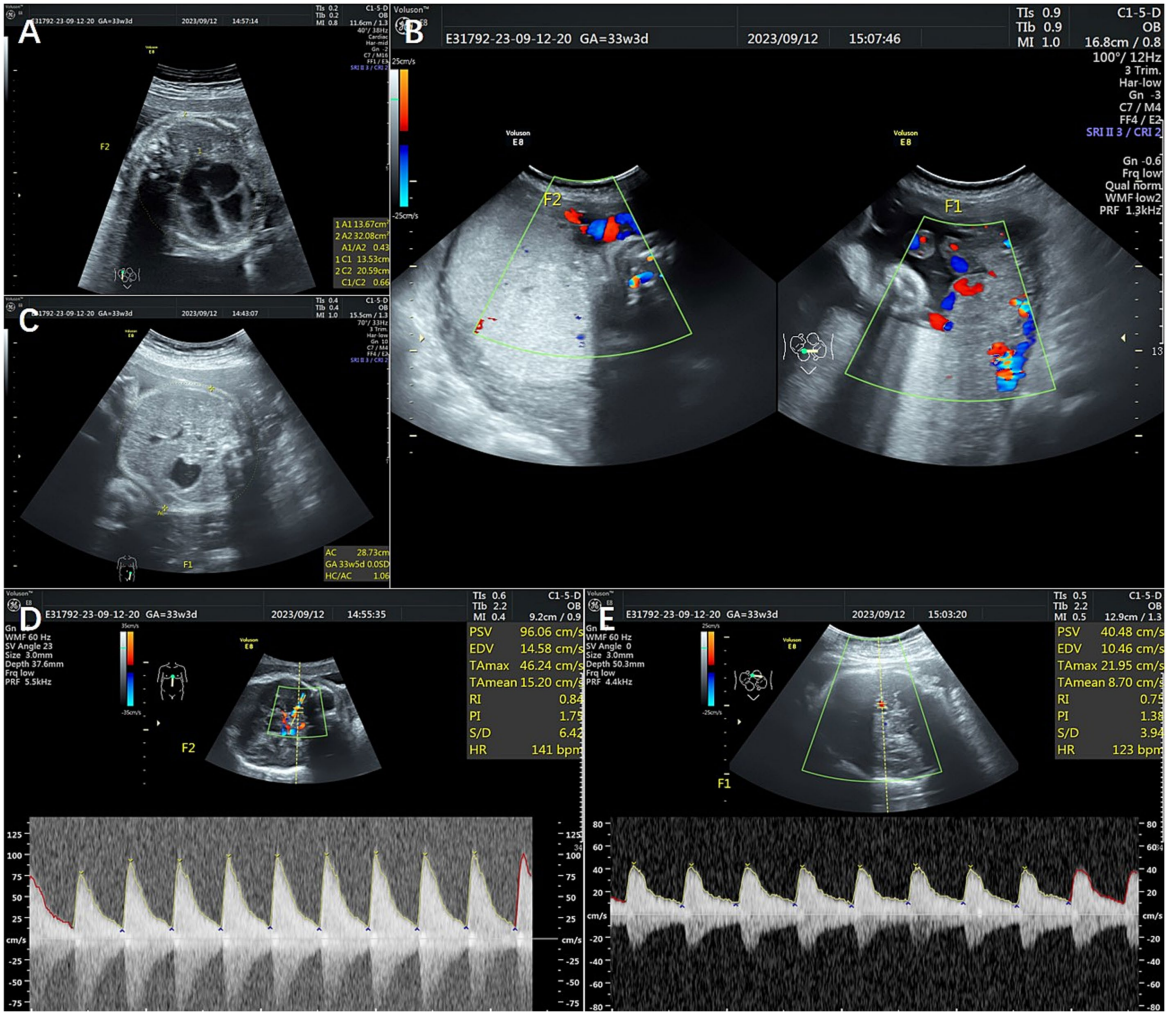
Ultrasonography of Patient 1. **(A)** Shows an increased cardiothoracic ratio of 0.66. **(B)** Shows the hyperechoic placenta in twin A and the thickened and hypoechoic placenta in twin B. **(C)** Shows a starry sky liver. **(D,E)** Show abnormal middle cerebral artery peak systolic velocities (MCA-PSVs) in both twins. F1 shows twin A, and F2 shows twin B.

A 34-year-old multipara, gravida 5 para 2, had a monochorionic diamniotic twin (MCDA) pregnancy. Her previous routine checks were normal. She was admitted to the hospital after positive ultrasound findings at 33 weeks of gestation. The ultrasound revealed abnormal middle cerebral artery peak systolic velocities (MCA-PSVs) in both twins (twin A: 0.86 multiples of the median (MoM) and twin B: 2.0 MoM), combined with a starry sky liver and hyperechoic placenta in twin A (recipient) and a thickened and hypoechoic placenta, an increased fetal cardiothoracic ratio, and an increased intravenous catheter pulsatility index in twin B (donor); the amniotic fluid volumes were 35 and 30 mm, respectively, indicating a stage 3 TAPS (Figure 1). After discussion between the multidisciplinary medical team and the family and the completion of antenatal glucocorticoid treatment, a cesarean section was performed. The newborns weighing 1900 and 1,250 grams were transferred to the neonatal intensive care unit (NICU). The hemoglobin level was 218 g/dL in the recipient twin and 49 g/dL in the donor twin. Moreover, the reticulocyte counts were 0.284 × 10^12^/L and 0.294 × 10^12^/L, respectively. The donor twin received four small doses of red blood cell transfusion after birth and gradually reached a normal weight. No large communicating vessels were observed in the vascular junctions of the placenta via macroscopic pathology. The umbilical cord insertion points were at the opposite edge of the placenta. Histopathological examination revealed that immature intermediate villi dominated the placental part of fetus B; some villi were edematous, and distal villi were missing. Unfortunately, a vascular injection of the placenta was not performed. Both twins had normal development at 1 year after birth.

### Patient 2

**Figure 2:**
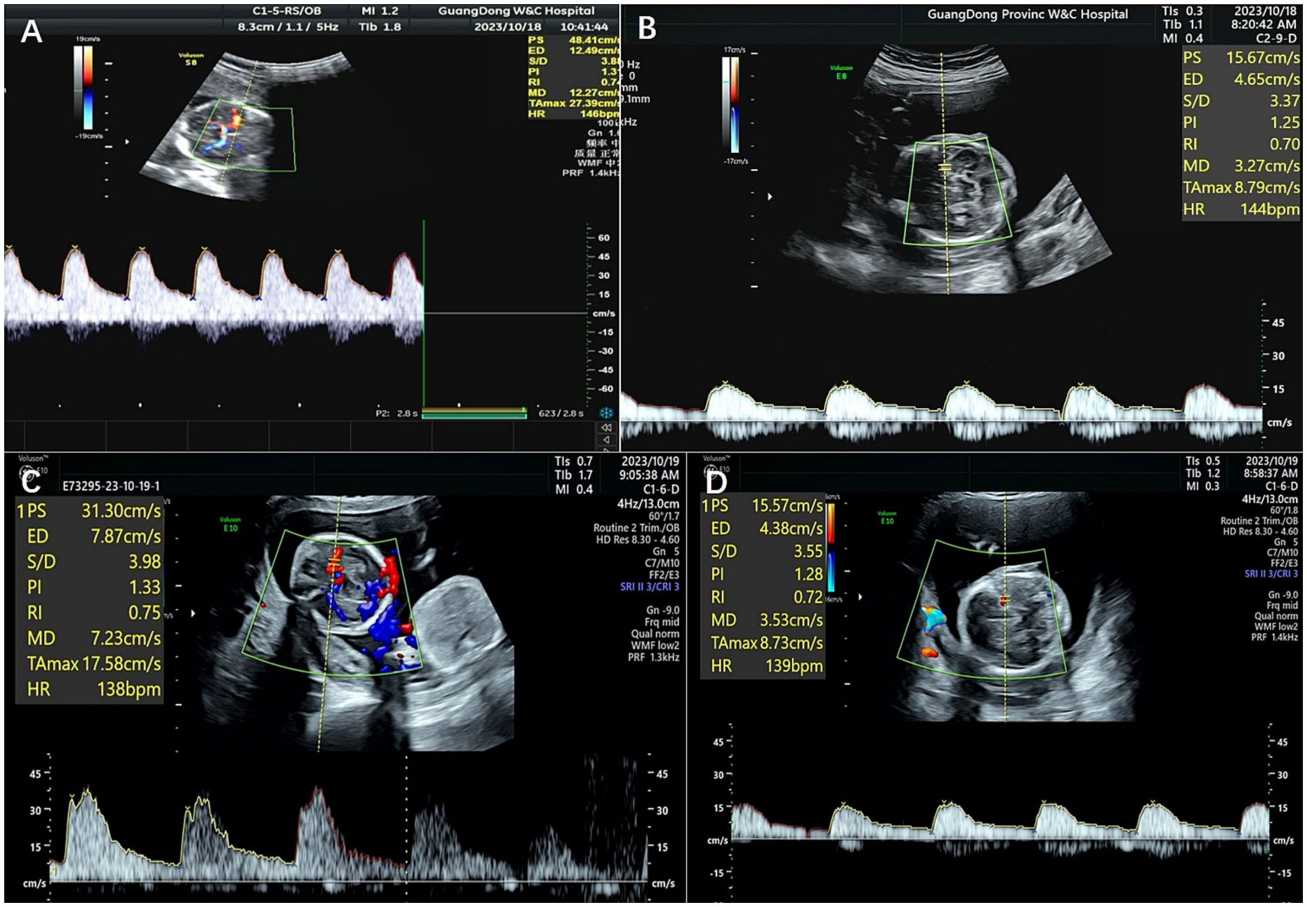
MCA-PSVs on ultrasonography of the fetuses of Patient 2 before and after laser surgery. **(A,B)** Show MCA-PSVs of the twins before laser surgery, and **(C,D)** show MCA-PSVs of the twins after surgery.

**Figure 3:**
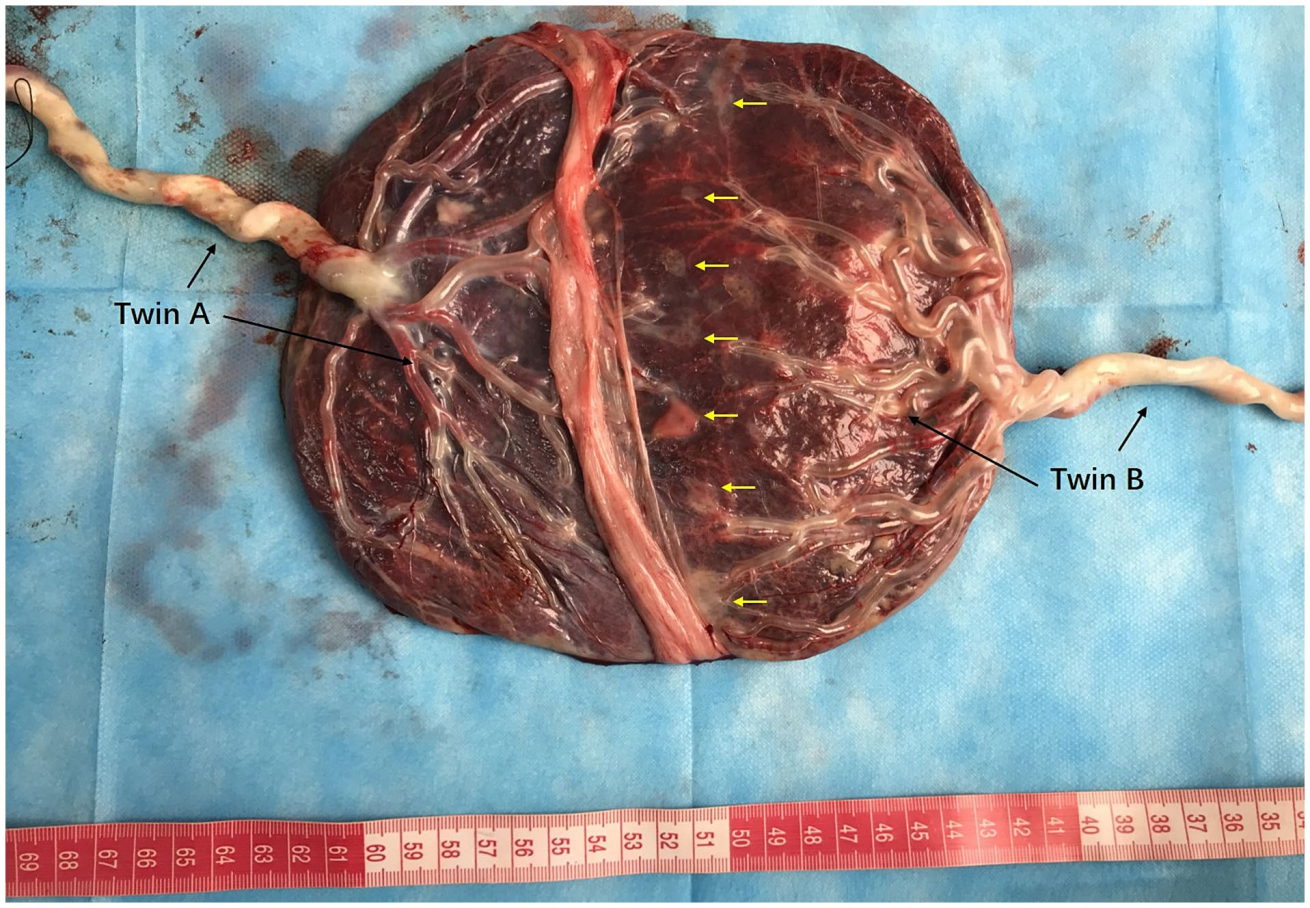
Placenta and umbilical vessels of Patient 2 after air infusion. The placental areas and diameters of the umbilical cords of twins A and B are shown. The vessels were separated from each other between the junctional parts of the placenta when air was sequentially infused into the umbilical arteries. Traces of laser cautery of blood vessels were clearly observed (yellow arrows). The battledore placenta of twin B.

The patient was a nulliparous 34-year-old woman who conceived naturally. An MCDA pregnancy was confirmed at 13 weeks of gestation. During a routine antenatal check at 18 weeks of gestation, an ultrasound revealed an increased MCA-PSV of 1.81 MoM in one fetus and a decreased MCA-PSV of 0.72 MoM in the other fetus, which indicated a stage 2 TAPS. The patient was referred to the Guangdong Women and Children Hospital for further treatment. A systemic structured ultrasound was performed. Twin A was the recipient, with an MCA-PSV of 0.66 MoM, choroid plexus cysts and a starry sky liver. Twin B was the donor, with an MCA-PSV of 2.05 MoM (Figures 2A,B), an enlarged heart, a cardiothoracic ratio of 0.55, a thickened placenta of 46.8 mm, and enhanced placental echogenicity; the amniotic fluid volume was 72 mm in the donor sac and 62 mm in the recipient sac; the weight difference between the twins was 30.8%, which also indicated complicated selective intrauterine growth restriction (sIUGR). The patient underwent fetoscopic laser placental communication vascular coagulation surgery at 18 weeks plus 2 days after the assessment, especially because of the early gestational age and the possibility of disease aggravation. The procedure was performed after local anesthesia and under ultrasound monitoring and guidance. The puncture approach for entering the trocar was chosen from the amniotic cavity of the donor because the donor fetus had a battledore placenta and the umbilical cord insertion point was far from the puncture point. We infused 300 mL of Ringer’s solution into the amnion and coagulated seven main A-V anastomoses, and amniotic fluid was reduced by 400 mL during the operation. An ultrasound performed 24 h following the procedure revealed a significant decrease in the MCA-PSV of the donor twin (Figures 2C,D). Together, genetic tests of the amniotic cells performed during surgery revealed no positive results. After discharge, regular ultrasonography monitoring was scheduled, the MCA-PSVs were stable, and sIUGR improved gradually (Table 1). The ultrasound interval was 2 weeks before 28 weeks of gestation and 1 week thereafter because of sIUGR of twin B. A cesarean section was subsequently performed because of spontaneous preterm birth at 32 + 2 weeks of gestation. The newborns weighing 1800 and 1,530 grams were transferred to the NICU immediately after birth. There were no significant differences in their hemoglobin values after birth. The placenta was examined after delivery, and the placental area of twin B was relatively smaller than that of twin A. In addition, the umbilical cord diameter of twin B was less than that of twin A. Traces of blood vessel laser cautery were clearly observed at the junction between the two umbilical vascular systems. The vessels (arteries of one twin and veins of the other) were separated from each other at the junction when air was sequentially infused into the two pairs of umbilical arteries (Figure 3). A histopathological examination revealed no significant abnormalities. The two newborns reached their normal development milestones in the months following discharge.

**Table 1:** Ultrasound monitoring results of Patient 2 before and after fetoscopic laser surgery.

Weeks+days	Twin	BPD (mm)	HC (mm)	AC (mm)	FL (mm)	AFV (mm)	EFW (g)	Weight difference	UA-S/D	MCA-PSV (cm/s)	MCA-PSV (MoM)	Placenta thickness (mm)	Cardiothoracic ratio
18	A	41	154	129	27	44	232	24.14%	3.7	16.76	0.72	19.3	
	B	38	135	110	24	47	176		4.1	42.22	1.81	51.4	
18 + 2	A	44	155	130	28	75	250	30.8%	3.05	15.67	0.66	16	0.39
	B	39	142	111	22	65	173		3.3	48.41	2.05	46.8	0.55
18 + 3	A									15.57	0.66		
	B									31.3	1.32		
19	A									14.76	0.61		
	B									32.24	1.32		
20 + 1	A	49	182	159	34	56	385	28.83%	3.65	16.69	0.65	20	
	B	44	159	141	28	57	274		3.9	24.62	0.96	37	
21 + 3	A	53	192	171	37	65	465	28.39%	4.67	20	0.74	26	0.27
	B	48	179	157	30	51	333		3.83	24.4	0.90	34	0.28
22 + 3	A	55	199	176	40	46	513	19.3%	3.11	23.9	0.84	27	
	B	51	187	166	35	49	414		4.83	32.69	1.15	36	
23 + 3	A	60	217	185	44	41	636	28.77%	3.24	26.9	0.90	18	
	B	54	202	169	37	50	453		3.43	36.4	1.22	35	
25 + 3	A	65	235	197	48	35	787	25.41%	3.11	28.5	0.87		
	B	60	221	190	39	51	587		3.04	39.8	1.22		
27 + 3	A	70	250	224	52	51	1,025	21.07%	3.42	32.5	0.90		
	B	65	241	205	47	47	809		2.51	42.8	1.19		
28 + 3	A	74	262	233	52	61	1,138	15.29%	2.67	40	1.06		
	B	69	252	226	47	46	964		2.93	43.4	1.15		
29 + 3	A	76	264	238	56	42	1,286	20.14%	3.46	46.4	1.18	13	
	B	71	251	224	51	40	1,027		3.1	41	1.04	39	
30 + 3	A	79	279	256	58	37	1,530	14.71%	2.6	43.35	1.05	23	0.32
	B	74	273	250	53	59	1,305		3.01	57.98	1.41	52	0.34
31 + 3	A	79	284	262	58	59	1,563	8.38%	2.29			23	0.35
	B	76	277	259	54	57	1,432		3.06			52	0.35

BPD, biparietal diameter; HC, head circumference; AC, abdominal circumference; FL, femur length; AFV, amniotic fluid volume; EFW, estimated fetal weight; UA-S/D, umbilical artery-systolic/diastolic ratio; MCA-PSV, middle cerebral artery peak systolic velocity; MoM, multiples of the median.

## Discussion

The two cases of TAPS reported herein both involved spontaneous TAPS; one was discovered in the third trimester, and the other was discovered in a relatively early stage of pregnancy. According to the consensus in the research on TAPS (7–9), antenatal diagnostic criteria for TAPS should be an MCA-PSV >1.5 MoM in the donor and an MCA-PSV <1.0 MoM in the recipient, and postnatal diagnosis is made by the measurement of an intertwin hemoglobin difference of ≥8.0 g/dL, in conjunction with an intertwin reticulocyte ratio (reticulocyte count of the donor twin divided by the reticulocyte count of the recipient twin) of >1.7 or a placenta with only small (diameter < 1 mm) vascular anastomoses. However, our data from Patient 1 did not reveal a difference in the reticulocyte counts between the cotwins after birth. An examination of the placenta was subsequently performed to determine whether there were vascular anastomoses and what type of vascular anastomoses it exactly was. To explore very small superficial A–V anastomoses, routine placental vessel perfusion in monochorionic twin pregnancies is recommended after delivery (10).

Regularly monitoring the MCA-PSVs of the cotwins via ultrasound is the most important method for evaluating the fetal conditions after the diagnosis of TAPS (11). The monitoring interval should be 1 to 2 weeks due to different fetal conditions. The fetal weight is another factor that should be focused upon in ultrasound observations; sIUGR is a complication that occurs in monochorionic twins. The umbilical artery end-diastolic flow spectrum is another key monitoring indicator. Monitoring the amniotic fluid volume in monochorionic twins can help identify acute complications such as TTTS.

The optimal management of TAPS differs among patients. Conservative management, fetoscopic laser coagulation, selective twin reduction, fetal blood exchange and transfusion, and delivery may be selected considering the gestational age at diagnosis, the severity of the condition, the likelihood of success, and the patients’ priorities (12). Conservative management is considered when the progression of the MCA-PSVs of the twins is stable. Selective fetal reduction is safe but is chosen only when there is a significant disparity in the MCA velocities of the twins or when one cotwin has structural or development abnormalities. An intrauterine transfusion (IUT) of red blood cells to the anemic fetus can be performed in chronic cases of TAPS. Repeat procedures are based on subsequent MCA-PSVs. An intraperitoneal transfusion is considered as a sole procedure or can be combined with IUT because it can allow for the slow absorption of red blood cells (13, 14). Fetal blood exchange uses equal volumes of sterile saline to remove certain aliquots of fetal blood in some cases. This procedure potentially reduces the complications associated with hyperviscosity. Gestational age is a crucial factor in the management of TAPS. If TAPS is found in the third trimester, especially after 32 weeks, delivery or expectant observation should be considered according to the fetal conditions.

Considering that conditions may be aggravated during long pregnancy periods, it is generally necessary to manage early-trimester TAPS via surgical procedures such as fetoscopic laser therapy (1, 4). Fetoscopic laser surgery is the only curative treatment but is more challenging in TAPS than in routine TTTS procedures because of the absence of polyhydramnios, which requires the injection of saline or Ringer’s solution through the amniotic membrane to improve visibility. Accurately locating tiny arteriovenous anastomoses and performing laser coagulation are other key challenges of the procedure because of the small and inconspicuous vascular anastomoses. Complications such as chorioamniotic membrane separation, miscarriage and preterm prelabor rupture of membranes should be closely monitored after the procedure (15).

Follow-up data, especially neurodevelopmental outcomes, should be recorded. Since the mechanism of TAPS is not clear, the collection of such information can be beneficial for future studies.

## Data Availability

The raw data supporting the conclusions of this article will be made available by the authors, without undue reservation.
